# Anti-Severe Acute Respiratory Syndrome Coronavirus 2 Immunoglobulin G Antibody Seroprevalence Among Truck Drivers and Assistants in Kenya

**DOI:** 10.1093/ofid/ofab314

**Published:** 2021-06-12

**Authors:** E Wangeci Kagucia, John N Gitonga, Catherine Kalu, Eric Ochomo, Benard Ochieng, Nickline Kuya, Angela Karani, James Nyagwange, Boniface Karia, Daisy Mugo, Henry K Karanja, James Tuju, Agnes Mutiso, Hosea Maroko, Lucy Okubi, Eric Maitha, Hossan Ajuck, David Mukabi, Wycliffe Moracha, David Bulimu, Nelson Andanje, Rashid Aman, Mercy Mwangangi, Patrick Amoth, Kadondi Kasera, Wangari Ng’ang’a, Amek Nyaguara, Shirine Voller, Mark Otiende, Christian Bottomley, Charles N Agoti, Lynette I Ochola-Oyier, Ifedayo M O Adetifa, Anthony O Etyang, Katherine E Gallagher, Sophie Uyoga, Edwine Barasa, Philip Bejon, Benjamin Tsofa, Ambrose Agweyu, George M Warimwe, J Anthony G Scott, The Magarini Sub-County TDA SARS-CoV-2 Serosurveillance Team, The Busia County TDA SARS-CoV-2 Serosurveillance Team

**Affiliations:** 1 KEMRI-Wellcome Trust Research Programme, Kilifi, Kenya; 2 KEMRI Centre for Global Health Research (CGHR), Kisumu, Kenya; 3 KEMRI Centre for Infectious and Parasitic Diseases Control Research, Busia, Kenya; 4 Department of Health, Kilifi County, Kenya; 5 Department of Health, Busia County, Kenya; 6 Ministry of Health, Government of Kenya, Nairobi, Kenya; 7 Presidential Policy and Strategy Unit, The Presidency, Government of Kenya, Nairobi, Kenya; 8 Department of Infectious Disease Epidemiology, London School of Hygiene and Tropical Medicine, London, United Kingdom; 9 Nuffield Department of Medicine, Oxford University, Oxford, United Kingdom

**Keywords:** antibody seroprevalence, frontline workers, Kenya, SARS-CoV-2, truck drivers

## Abstract

In October 2020, anti-severe acute respiratory syndrome coronavirus 2 (SARS-CoV-2) *immunoglobulin* G seroprevalence among truck drivers and their assistants (TDA) in Kenya was 42.3%, higher than among healthcare workers and blood donors. Truck drivers and their assistants transport essential supplies during the coronavirus disease 2019 pandemic, placing them at increased risk of being infected and of transmitting SARS-CoV-2 over a wide geographical area.

Serosurveillance for antibodies to the severe acute respiratory syndrome coronavirus 2 (SARS-CoV-2) can be used to estimate the cumulative incidence of SARS-CoV-2 infection. This is of particular interest among frontline workers, such as truck drivers, who are exempted from coronavirus disease 2019 (COVID-19) restrictions such as curfews and lockdowns.

Shortly after the first locally detected SARS-CoV-2 infection in March 2020, the Government of Kenya instituted containment measures, including travel restrictions. Freight transportation was excluded from travel restrictions because truckers and drivers’ assistants (TDA) were deemed essential workers [1]. In May 2020, the Kenya Ministry of Health implemented mandatory bimonthly SARS-CoV-2 nucleic acid testing (NAT) for long-distance TDA, in line with East African Community guidelines [2, 3]. Although NAT data can be used to estimate the cumulative incidence of SARS-CoV-2 infection [4], accurate estimates are dependent on the availability of reliable model parameter values.

There are currently no estimates of SARS-CoV-2 antibody seroprevalence among TDA globally. Due to the nature of their work, TDA interact with a wide variety of social and professional contacts over a large geographical area, putting them at increased risk of SARS-CoV-2 infection. We undertook a serosurvey to estimate the prevalence of SARS-CoV-2 antibodies among TDA in Kenya.

## METHODS

### Study Design and Participants

A cross-sectional serosurvey was conducted at 3 sites: 1 in Kilifi County in South-Eastern Kenya and 2 in Busia County in Western Kenya (Supplementary Figure 1). In Kilifi County, TDA transporting salt domestically and to East African countries (eg, Uganda, Rwanda and Burundi) were engaged at salt harvesting companies located in Gongoni area, Magarini Sub-County. In Busia County, TDA were engaged at One Stop Border Posts (OSBPs) in 2 border towns, Busia and Malaba. Truckers and drivers’ assistants at Busia OSBP and Malaba OSBP typically transport freight between Mombasa, Kenya and countries in East Africa (Supplementary Figure 2).

Truckers and drivers’ assistants were eligible if they were aged ≥18 years without a medical contraindication for blood sample collection. Participation was voluntary and verbal consent was sought before blood sample collection. Serosurveillance was conducted as a public health activity requested by the Kenya Ministry of Health, and ethical approval for publication of these data was obtained from the Kenya Medical Research Institute Scientific and Ethics Review Unit (KEMRI/SERU/CGMR-C/203/4085).

### Patient Consent Statement

The study did not include factors necessitating patient consent.

### Data and Sample Collection

Sociodemographic and clinical data, including age, sex, nationality, residence, temperature, and symptoms of illness within the previous 2 weeks, were collected by county public health staff (CPHS) and recorded on governmental COVID-19 surveillance forms (Supplementary Forms 1 and 2). Venous blood samples were collected using standard blood sample collection procedures and labeled with the same unique identifier as nasopharyngeal/oropharyngeal (NP/OP) samples. The NP/OP NAT results, linked using the unique identifiers, were obtained from testing laboratories. Data with personal identifiers were retained only by CPHS.

### Laboratory Testing

Plasma was extracted from blood samples using standard techniques and stored at −80°C before antibody testing. Thawed plasma samples were tested for anti-SARS-CoV-2 *immunoglobulin* G (IgG) using a locally validated whole spike enzyme-linked immunosorbent assay (ELISA) with 93% sensitivity and 99% specificity, as described previously [5, 6]. Spike ELISA positivity was defined as a ratio of sample optical density (OD) over negative control OD >2. The NP/OP NAT results are routinely provided to TDA by CPHS. Serology results were also provided to TDA by CPHS.

### Statistical Analysis

At least 700 samples were required to estimate seroprevalence as low as 2% within a 1% margin of error. Age was categorized into 10-year strata. Crude seroprevalence was estimated as the number of anti-SARS-CoV-2 IgG-positive samples (OD ratio >2) divided by all samples, and associated exact binomial 95% confidence intervals (CIs) were calculated. Bayesian adjustment for assay sensitivity and specificity was performed as previously described [6]. Associations between seropositivity and sociodemographic/clinical characteristics were tested using χ ^2^ or Fisher’s exact tests. Test-performance-adjusted seroprevalence and credible intervals were calculated in R (version 3.6.1) with RStan. All other analyses were performed using Stata (version 15.1).

## RESULTS

The serosurvey was conducted in Magarini between September 30 and October 23, 2020 and in Busia County on October 13–15, 2020. In total, 830 TDA (Busia OSBP n = 365; Magarini n = 101; Malaba OSBP n = 364) provided a blood sample, representing 52.4% of TDA approached (Supplementary Figure 3). Of the 830 samples, 91.1% were collected between October 13 and 15, 2020 (Supplementary Figure 4). The median age of sampled TDA was 40 years (interquartile range, 34–48 years) and 668 (80.5%) were Kenyan. Only 3 (0.4%) of the TDA were female (Table 1). Among Kenyan TDA, reported county of residence included 31 of the country’s 47 counties. The most common counties of residence were Mombasa, Uasin Gishu, and Nakuru (Supplementary Table 1). Driver versus assistant role was not recorded, but, on the basis of truck driver licensure age requirements, at least 53 (6.6%) were assistants (Table 1).

**Table 1.** Crude and Test-Performance Adjusted Anti-SARS-CoV-2 IgG Seroprevalence Among TDA in Kenya, September 30 to October 23, 2020

Characteristic	N	Seropositive	%Crude anti-SARS-CoV-2 IgG Seroprevalence	95% Confidence Interval	%Adjusted Anti-SARS-CoV-2 IgG Seroprevalence	95% Credible Interval
Total	830	329	39.6	36.3–43.1	42.3	38.4–46.3
Age^a^						
<30 years	92	42	45.7	35.2–56.4	49.1	38.1–59.9
30–39 years	276	110	39.9	34.0–45.9	42.4	35.5–49.1
40–49 years	286	116	40.6	34.8–46.5	43.4	37.4–49.9
50–59 years	135	48	35.6	27.5–44.2	38.1	29.2–47.4
>60 years	38	13	34.2	19.6–51.4	37.0	22.3–54.1
Sex						
Male	827	326	39.4	36.1–42.8	42.1	38.1–46.7
Female	3	3	100.0	29.2–100.0	79.6	37.9–99.3
NAT Result^a^						
Positive	58	32	55.2	41.5–68.3	59.0	46.0–72.1
Negative	727	270	37.1	33.6–40.8	39.6	35.0–43.9
Nationality						
Kenya	668	262	39.2	35.5–43.0	41.9	37.7–46.0
Other	162	67	41.4	33.7–49.4	44.2	36.3–52.5
Site						
Busia OSBP	365	163	44.7	39.5–49.9	47.9	42.3–53.8
Magarini	101	43	42.6	32.8–52.8	45.2	35.1–55.9
Malaba OSBP	364	123	33.8	28.9–38.9	36.0	30.8–41.9

Abbreviations: IgG, *immunoglobulin G*; NAT, nucleic acid test; OSBP, One Stop Border Post; SARS-CoV-2, severe acute respiratory syndrome coronavirus 2; TDA, truck drivers and assistants.

^a^Missingness: age, n = 3; NAT result, n = 45.

Of 785 TDA with available NAT results, 58 (7.4%) were positive for SARS-CoV-2 (Table 1); positivity was significantly higher in Magarini (25 of 93; 26.9%) compared to Busia OSBP (15 of 348; 4.3%) and Malaba OSBP (18 of 344; 5.2%; *P* < .001) (Supplementary Table 2). None of the 830 TDA reported current or previous symptoms of illness. Only 1 individual had a temperature of 37.5°C or higher (Supplementary Table 2) and the NAT result was negative in that 1 case.

Crude overall anti-SARS-CoV-2 IgG seroprevalence among sampled TDA was 39.6% (95% CI, 36.3−43.1). After adjustment for assay sensitivity and specificity it was 42.3% (95% credible interval [CrI], 38.4−46.3). Seroprevalence was highest in TDA <30 years of age and lowest among those aged ≥50 years. All 3 female TDA were seropositive for anti-SARS-CoV-2 IgG. Seroprevalence was 39.6% (95% CrI, 35.0−43.9) among TDA negative for SARS-CoV-2 by NAT and 59.0% (95% CrI, 46.0−72.1) among those with a positive SARS-CoV-2 NAT result. Seroprevalence among Kenyan TDA was comparable to that of other nationalities. By site, seroprevalence was highest at Busia OSBP and lowest at Malaba OSBP. Crude heterogeneity tests and uncertainty intervals for antibody test-adjusted estimates suggested no significant association between seroprevalence and age and significant differences in seroprevalence by site (Table 1 and Supplementary Table 3).

## Discussion

We estimate that approximately 4 in 10 TDA in Kenya had been infected with SARS-CoV-2 by late October 2020, as the country experienced the initial stages of a second pandemic wave (Supplementary Figure 5). This study is notable for being the first to report SARS-CoV-2 seroprevalence among TDA.

Anti-SARS-CoV-2 IgG seroprevalence among TDA was more than 4-fold higher compared to average seroprevalence among Kenyan blood donors by September 2020 [7] and 2-fold higher compared to average seroprevalence in healthcare workers (HCWs) across 5 Kenyan health facilities by October 2020 [8]. As such, the findings suggest that the cumulative incidence of SARS-CoV-2 among TDA in Kenya was higher than in the general population and in other frontline workers.

Outside Kenya, some studies found 44%−45% seroprevalence of SARS-CoV-2 antibodies among HCWs, comparable to that among TDA in Kenya [9–11]. However, meta-analyses of studies globally estimated SARS-CoV-2 antibody prevalence among HCWs at 7% by July 2020 [12] and 9% by August 2020 [13]. Taken together, these data suggest that exposure to SARS-CoV-2 among TDA was higher than among HCWs in most settings.

To date, few studies have assessed SARS-CoV-2 antibody prevalence in nonhealthcare frontline workers. Seroprevalence was 38% seroprevalence among male migrant supermarket workers in Kuwait in May to June 2020 [14]. Elsewhere, however, seroprevalence among non-HCWs has been lower, ranging from 1% [15] to 22% [16].

None of the NAT-positive TDA reported symptoms within 2 weeks of testing, suggesting predominantly asymptomatic COVID-19 among TDA. Although data on symptoms may have been subject to reporting bias, 93% of SARS-CoV-2 infections confirmed in Kenya by October 23, 2020 were asymptomatic [17]. The similarity in anti-SARS-CoV-2 antibody seropositivity among Kenyan and non-Kenyan TDA indicates predominantly occupation-related exposure to SARS-CoV-2. The fact that seroprevalence differed by sampling site suggests some heterogeneity in exposure risk.

Despite sampling at 3 different sites, we cannot be sure that the sample included are representative of all TDA in Kenya, or even those transiting through these sites. Participation in the serosurvey was voluntary, only half of those approached agreed to be sampled, and sociodemographic data for the target population are not available. However, data on county of residence for Kenyan TDA indicates representation from most counties. Seroprevalence estimates did not account for clustering by site or by driver-assistant pairs. The use of antibodies to estimate cumulative incidence of SARS-CoV-2 infection assumes universal seroconversion, minimal mortality, and antibody persistence. If these assumptions are incorrect—for example, there is evidence suggestive of antibody waning [18–20]—the cumulative incidence of infection would be underestimated. To minimize bias due to assay sensitivity and specificity, adjusted seroprevalence estimates were calculated.

## Conclusions

These findings indicate substantial cumulative incidence of SARS-CoV-2 infection—most of which was asymptomatic—among TDA in Kenya, despite prevention measures. The occupational nature of haulage entails interaction with many contacts over large geographical areas; as such, TDA may unintentionally contribute to the spread of emerging infections. These findings illustrate the challenge of infection prevention within an occupational group whose role in sustaining supply chains remains vital during a pandemic. In addition, they underscore the importance of national and regional preparedness plans for the safe continuation of haulage in future pandemics.

## Supplementary Data

Supplementary materials are available at *Open Forum Infectious Diseases* online. Consisting of data provided by the authors to benefit the reader, the posted materials are not copyedited and are the sole responsibility of the authors, so questions or comments should be addressed to the corresponding author.

ofab314_suppl_Supplementary_Material
