# Supplementary material for: Anti-Severe Acute Respiratory Syndrome Coronavirus 2 Immunoglobulin G Antibody Seroprevalence Among Truck Drivers and Assistants in Kenya
Source: Open Forum Infect Dis. 2021 Jun 12;8(7):ofab314. doi: 10.1093/ofid/ofab314 (PMC8519263; doi:10.1093/ofid/ofab314)
Supplement: ofab314_suppl_Supplementary_Material [file ofab314_suppl_supplementary_material.docx]

Supplement

Seroprevalence of anti-SARS-CoV-2 IgG antibodies among truck drivers and assistants in Kenya

E Wangeci Kagucia,^1^ John N Gitonga,^1^ Catherine Kalu,^1^ Eric Ochomo,^2^ Benard Ochieng,^2^ Nickline Kuya,^2^ Angela Karani,^1^ James Nyagwange,^1^ Boniface Karia,^1^ Daisy Mugo,^1^ Henry K Karanja,^1^ James Tuju,^1^ Agnes Mutiso,^1^ Hosea Maroko,^3^ Lucy Okubi,^3^ Eric Maitha,^4^ Hossan Ajuck,^4^ David Mukabi,^5^ Wycliffe Moracha,^5^ David Bulimu,^5^ Nelson Andanje,^5^ The Magarini Sub-County TDA SARS-CoV-2 Serosurveillance Team, The Busia County TDA SARS-CoV-2 Serosurveillance Team, Rashid Aman,^6^ Mercy Mwangangi,^6^ Patrick Amoth,^6^ Kadondi Kasera,^6^ Wangari Ng’ang’a,^7^ Amek Nyaguara,^1^ Shirine Voller,^1,8^ Mark Otiende,^1^ Christian Bottomley,^8^ Charles N Agoti,^1^ Lynette I Ochola-Oyier,^1^ Ifedayo M O Adetifa,^1,8^ Anthony O Etyang,^1^ Katherine E Gallagher,^1,8^ Sophie Uyoga,^1^ Edwine Barasa,^1^ Philip Bejon,^1,9^ Benjamin Tsofa,^1^ Ambrose Agweyu,^1*^ George M Warimwe,^1,9*^ J Anthony G Scott^1,8,9*^

^1^KEMRI-Wellcome Trust Research Programme, Kilifi, Kenya

^2^KEMRI Center for Global Health Research (CGHR), Kisumu, Kenya

^3^KEMRI Center for Infectious and Parasitic Diseases Control Research, Alupe, Kenya

^4^Department of Health, Kilifi County, Kenya

^5^Department of Health, Busia County, Kenya

^6^Ministry of Health, Government of Kenya, Nairobi, Kenya

^7^Presidential Policy and Strategy Unit, The Presidency, Government of Kenya

^8^Department of Infectious Disease Epidemiology, London School of Hygiene and Tropical Medicine, UK

^9^Nuffield Department of Medicine, Oxford University, UK

*These authors contributed equally to this work

Contents

[Members of the Magarini Sub-County TDA SARS-CoV-2 Serosurveillance Team 3](#_Toc72270321)

[Members of the Busia County TDA SARS-CoV-2 Serosurveillance Team 3](#_Toc72270322)

[Supplementary Table 1. Reported county of residence among Kenyan truckers 4](#_Toc72270323)

[Supplementary Table 2. Distribution of characteristics by site. 5](#_Toc72270324)

[Supplementary Table 3. Stratum-specific tests for heterogeneity using crude anti-SARS-CoV-2 IgG seroprevalence 6](#_Toc72270325)

[Supplementary Figure 1. Map of Kenya showing the serosurvey sites 7](#_Toc72270326)

[Supplementary Figure 2. Map showing the Northern Corridor road network. 8](#_Toc72270327)

[Supplementary Figure 3. Flow of TDA included in the SARS-CoV-2 serosurvey 9](#_Toc72270328)

[Supplementary Figure 4. Sampling by date and site 10](#_Toc72270329)

[Supplementary Figure 5. Daily confirmed COVID-19 cases in Kenya through the second pandemic wave. 11](#_Toc72270330)

[Supplementary Form 1 13](#_Toc72270331)

[Supplementary Form 2 16](#_Toc72270332)

[References 17](#_Toc72270333)

# Members of the Magarini Sub-County TDA SARS-CoV-2 Serosurveillance Team

Hossan Ajuck

Mary Bogita

Richmond Mudindi

Arthur Mwangi

Dorcas Mkanyi

George Karisa

Judith K Migosi

Patrick Msili

Samson Mwambire

Anthony M Boniface

# Members of the Busia County TDA SARS-CoV-2 Serosurveillance Team

David Mukabi

Wycliffe Moracha

David Bulimu

Nelson Andanje

Evans Shiraku

Rosemary Okuku

Monica Ogutu

Rodgers Mariga

Tobias Munabi

Susan M Ramadhan

Janet Mwikali

Rose Nasike

Cornelius Andera

Roselyne Nechesa

Benson K Kiplagat

Julius Omengo

Simon Oteba

# Supplementary Table 1. Reported county of residence among Kenyan truckers

| **County** | **Frequency** | **Percent** |  | **County** | **Frequency** | **Percent** |
| --- | --- | --- | --- | --- | --- | --- |
| MOMBASA | 156 | 23.4 |  | KIRINYAGA | 4 | 0.6 |
| UASIN GISHU | 76 | 11.4 |  | LAIKIPIA | 4 | 0.6 |
| NAKURU | 67 | 10.0 |  | SIAYA | 4 | 0.6 |
| NAIROBI | 60 | 9.0 |  | KITUI | 3 | 0.5 |
| KIAMBU | 41 | 6.1 |  | MANDERA | 3 | 0.5 |
| BUSIA | 40 | 6.0 |  | EMBU | 2 | 0.3 |
| MACHAKOS | 18 | 2.7 |  | KAJIADO | 2 | 0.3 |
| KWALE | 14 | 2.1 |  | SAMBURU | 2 | 0.3 |
| BUNGOMA | 13 | 2.0 |  | GARISSA | 1 | 0.2 |
| KAKAMEGA | 13 | 2.0 |  | HOMABAY | 1 | 0.2 |
| KISUMU | 13 | 2.0 |  | KISII | 1 | 0.2 |
| KERICHO | 8 | 1.2 |  | MIGORI | 1 | 0.2 |
| MAKUENI | 8 | 1.2 |  | TAITA TAVETA | 1 | 0.2 |
| TRANS NZOIA | 6 | 0.9 |  | *Uganda* | 1 | 0.2 |
| MURANGA | 5 | 0.8 |  | *Missing*^a^ | 91 | 13.7 |
| NYERI | 5 | 0.8 |  | Total | 668 | 100 |
| ELGEIYO MARAKWET | 4 | 0.6 |  |  |  |  |

^a^County of residence not available for 90 of 101 truckers sampled at Magarini due to differences in the data collection form used; County of residence not collected for 1 trucker sampled at Malaba

# Supplementary Table 2. Distribution of characteristics by site.

Tests for heterogeneity conducted using analysis of variance (age) or chi-squared tests (all other variables).

| **Characteristic** | **Busia OSBP**  **N= 365** | | **Magarini**  **N= 101** | | **Malaba OSBP**  **N= 364** | | **p-values** |
| --- | --- | --- | --- | --- | --- | --- | --- |
| **Mean age (SD), range**^a^ | 41.0y (9.6) | 20 – 68y | 38.4y (9.3) | 20 - 61y | 42.3y (9.9) | 19 – 78y | 0.002 |
| **Male, %** | 363 | 99.5% | 100 | 99.0% | 364 | 100% | 0.249 |
| **NAT positive, %**^b^ | 15 | 4.3% | 25 | 26.9% | 18 | 5.2% | <0.001 |
| **Kenyan, %** | 294 | 80.5% | 73 | 72.3% | 301 | 82.7% | 0.065 |
| **Symptoms, %** | 0 | 0% | 0 | 0% | 0 | 0% | N/A |
| **Temperature ≥37.5^0^C**^c^ | 0 | 0% | 0 | 0% | 1 | 0.3% | 0.547 |

Abbreviations: NAT, nucleic acid testing; OSBP, One Stop Border Post

^a^Missingness: Magarini = 2; Malaba = 1

^b^Missingness: Busia = 17; Magarini = 8; Malaba = 20

^c^Missingness: Busia = 12; Magarini = 17; Malaba = 1

# Supplementary Table 3. Stratum-specific tests for heterogeneity using crude anti-SARS-CoV-2 IgG seroprevalence

|  | **N** | **Seropositive** | **% Crude anti-SARS-CoV-2 IgG seroprevalence** | **95% CI** | **p-value** |
| --- | --- | --- | --- | --- | --- |
| **Age**^a^ |  |  |  |  |  |
| <30y | 92 | 42 | 45.7 | 35.2 – 56.4 | 0.144^a^ |
| 30-39y | 276 | 110 | 39.9 | 34.0 – 45.9 |  |
| 40-49y | 286 | 116 | 40.6 | 34.8 – 46.5 |  |
| 50-59y | 135 | 48 | 35.6 | 27.5 – 44.2 |  |
| >60y | 38 | 13 | 34.2 | 19.6 – 51.4 |  |
| **Sex** |  |  |  |  |  |
| Male | 827 | 326 | 39.4 | 36.1 – 42.8 | 0.062^b^ |
| Female | 3 | 3 | 100.0 | 29.2 – 100.0 |  |
| **NAT result** |  |  |  |  |  |
| Positive | 58 | 32 | 55.2 | 41.5 – 68.3 | 0.007 |
| Negative | 727 | 270 | 37.1 | 33.6 – 40.8 |  |
| **Nationality** |  |  |  |  |  |
| Kenya | 668 | 262 | 39.2 | 35.5 – 43.0 | 0.618 |
| Other | 162 | 67 | 41.4 | 33.7 – 49.4 |  |
| **Site** |  |  |  |  |  |
| Busia OSBP | 365 | 163 | 44.7 | 39.5 – 49.9 | 0.009 |
| Magarini | 101 | 43 | 42.6 | 32.8 – 52.8 |  |
| Malaba OSBP | 364 | 123 | 33.8 | 28.9 – 38.9 |  |

Abbreviations: NAT, nucleic acid test; OSBP, One Stop Border Post; SARS-CoV-2, severe acute respiratory syndrome coronavirus 2; TDA, truck drivers and assistants

^a^Chi-square test for trend performed

^b^Fisher’s exact test performed

# Supplementary Table 4. Acknowledgment list

| **KEMRI-Wellcome Trust Research Programme COVID-19 Laboratory Team** | |
| --- | --- |
| Isabella Lynette Oyier | Peter Mwaura |
| Charles Nyaigoti | Edwin Machanja |
| Jennifer Musyoki | Alfred Mwanzu |
| Caroline Ngetsa | Kelly Ominde |
| Horace Gumba | James Chemweno |
| Martin Mutunga | Isaac Musungu |
| Robinson Cheruiyot | Margaret Nunah |
| Clement Lewa | George Githinji |
| Elijah Gicheru | Zaydah de` Laurent |
| Calleb Odundo | Donwilliams Omuoyo |
| Arnold Lambisia | Khadija Said |
| John Mwita | Wanjiru Mburu |
| Joyce Nyiro | Jedidah Mwacharo |
| Victor Osoti | Debra Riako |
| Shadrack Mutua | Faith Marura |
| Johnstone Makale | Domtila Kimani |
| Brian Bartilol | Leonard Ndwiga |
| Brian Tawa | Boniface Gichuki |
| Wilson Gumbi | Josephine Naimani |
| Metrine Tendwa | Robert Musyimi |
| Yiakon Sein | Clara Karani |
| Wesley Cheruiyot | Moses Mosobo |
| Wilfred Nyamu Musyoki | Salim Mwarumba |
| Shadrack Mutua | James Tuju |
| Nicole Achieng | James Nyagwange |
| Kelvias Keter | Micah Nyanoti |
| Angela Karani | Willy Towett |
| Shaban Mwangi | Michael Opiyo |
| Rita Warui | Susan Njuguna |
| Metrine Tendwa | Fred Mitsanze |
| Lydia Nyamako | Alfred Mwakubia |
| Justine Getonto | Oscar Kai |
| Agnes Mutiso | Susan Wangui |
| Ephantus Pariken | David Kortok |
| Faith Gambo | Anthony Nzaro |
| Sharon Owour | Masha Maitha |
| David Ireri |  |
| **KEMRI-Wellcome Trust Research Programme COVID-19 Data Team** | |
| Edward Otieno | Sarah Baya |
| Patience Rehema | Nelson Ouma |
| Shadrack Mramba | Lawrence Babu |
| **KEMRI-Wellcome Trust Research Programme PCVIS Team** | |
| Donald Akech | Francis Kazungu |
| Nelson Kalama | Thomas Mwamuye |
| Stephen Mangi | Timothy Mwaringa |
| Victor Gari | Donald Deche |
| Chome Kabuka | Japhet Mwatua |
| Fredrick Mwamuye | Eddison Kumbo |
| **KEMRI-Wellcome Trust Research Programme Engagement Team** | |
| Noni Mumba | Juliet Odhiambo |
| Salim Mwalukore | Alex Njeru |
| Betty Kalama | Community Liaison Group (CLG) |

# Supplementary Figure 1. Map of Kenya showing the serosurvey sites


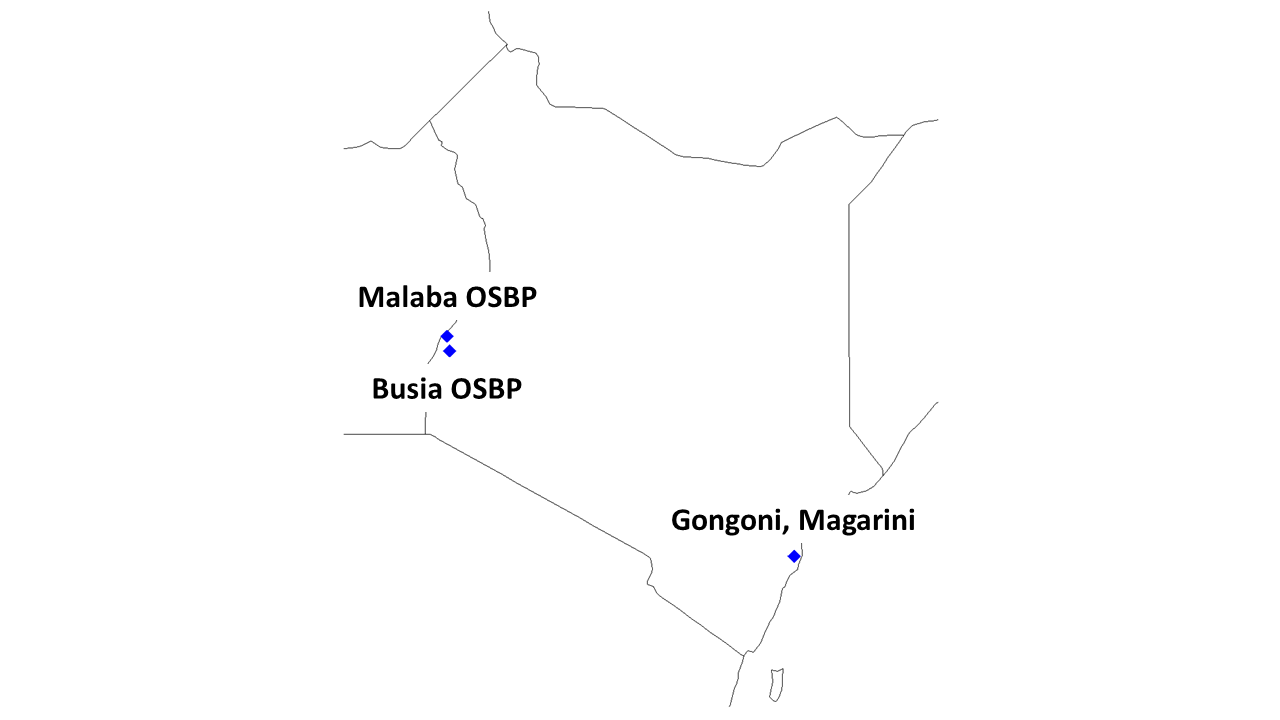


# Supplementary Figure 2. Map showing the Northern Corridor road network.

The Northern Corridor is used to transport freight from Mombasa, Kenya to other East African countries through Busia OSBP and Malaba OBSP, among other ports. Map from the Northern Corridor Transit and Transport Coordination Authority.^1^


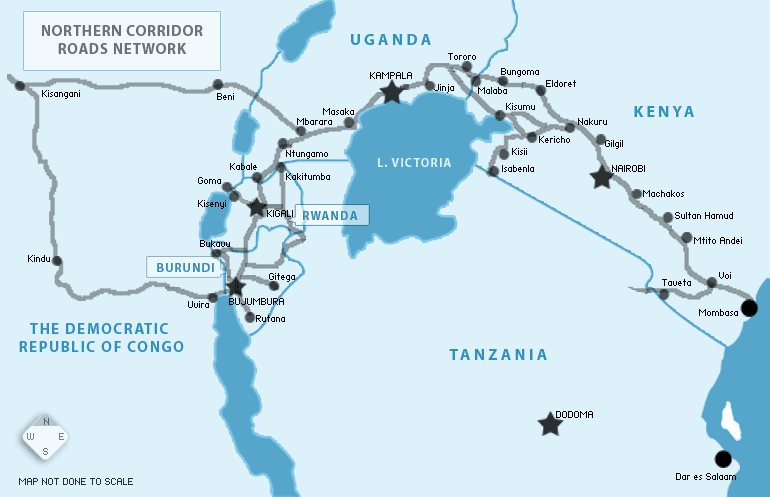


# Supplementary Figure 3. Flow of TDA included in the SARS-CoV-2 serosurvey


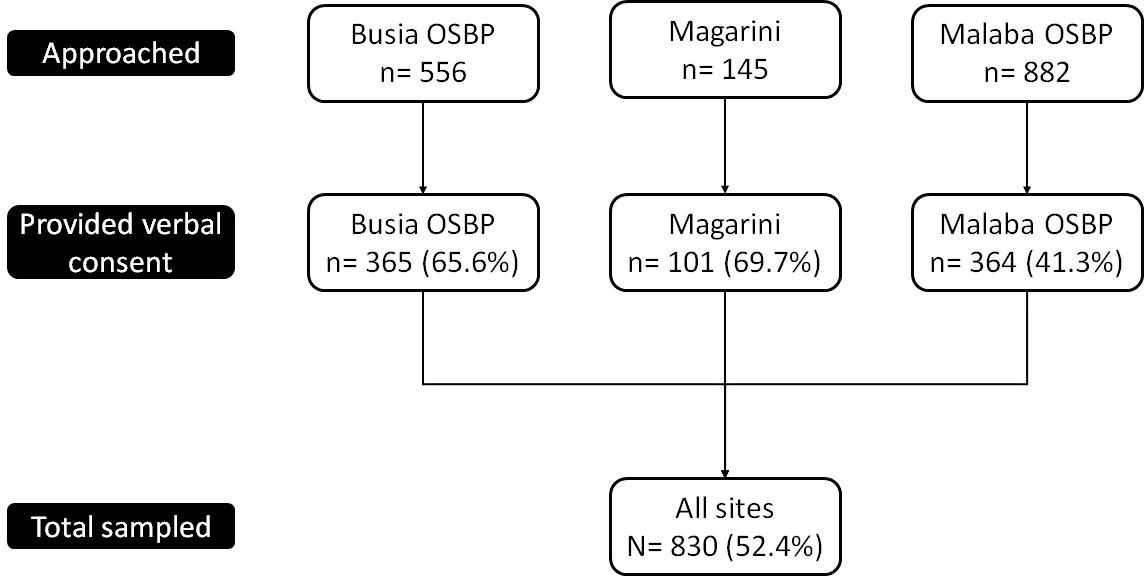


# Supplementary Figure 4. Sampling by date and site


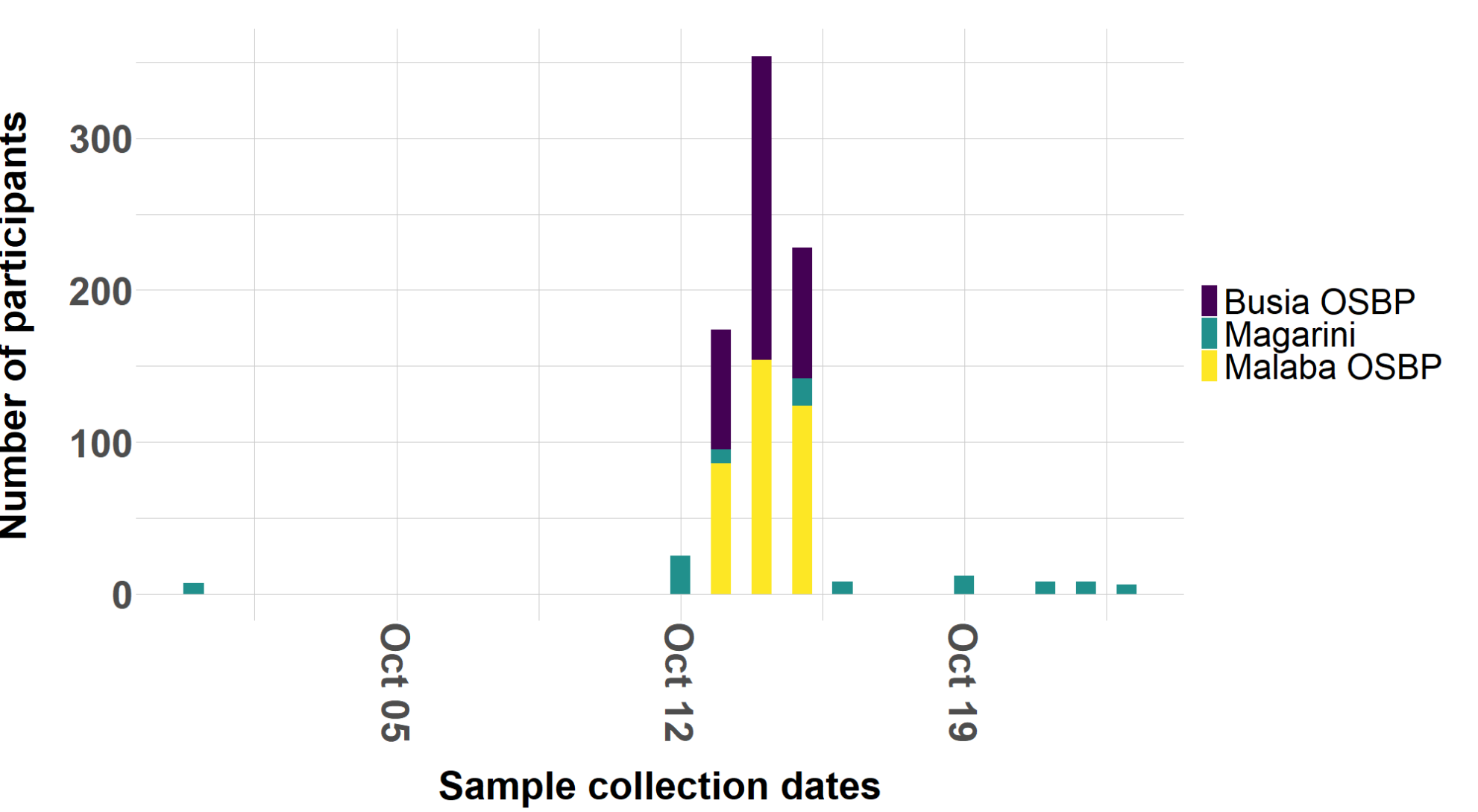


# Supplementary Figure 5. Daily confirmed COVID-19 cases in Kenya through the second pandemic wave.

The gray shaded area depicts the period during which the SARS-CoV-2 serosurvey among TDA was conducted. Data from Ritchie et al.^2^

# Supplementary Form 1


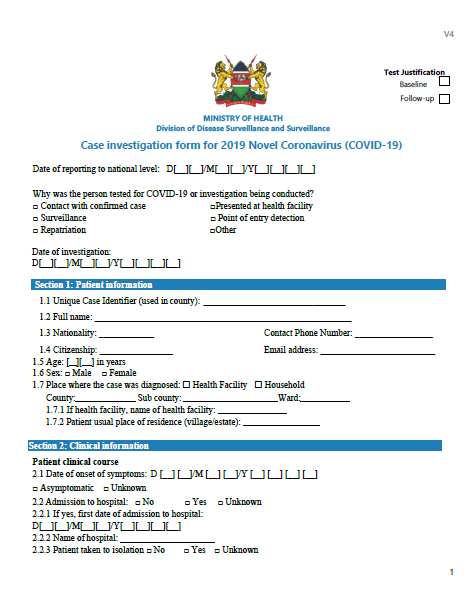


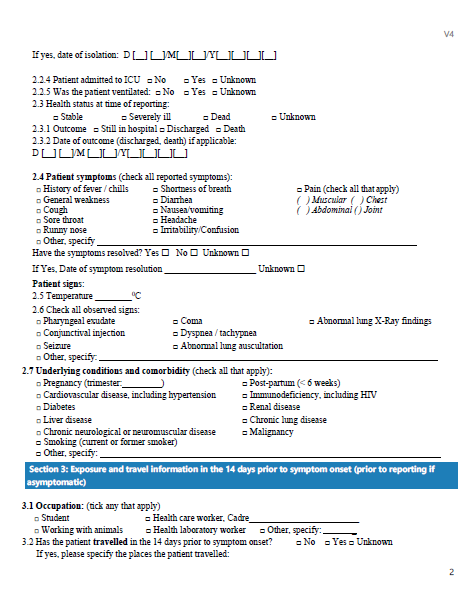


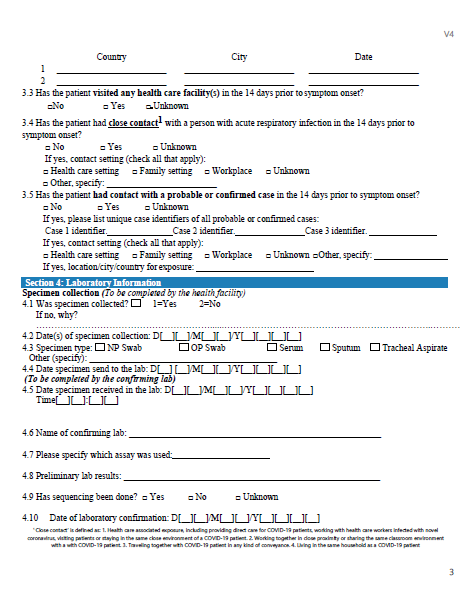


# Supplementary Form 2


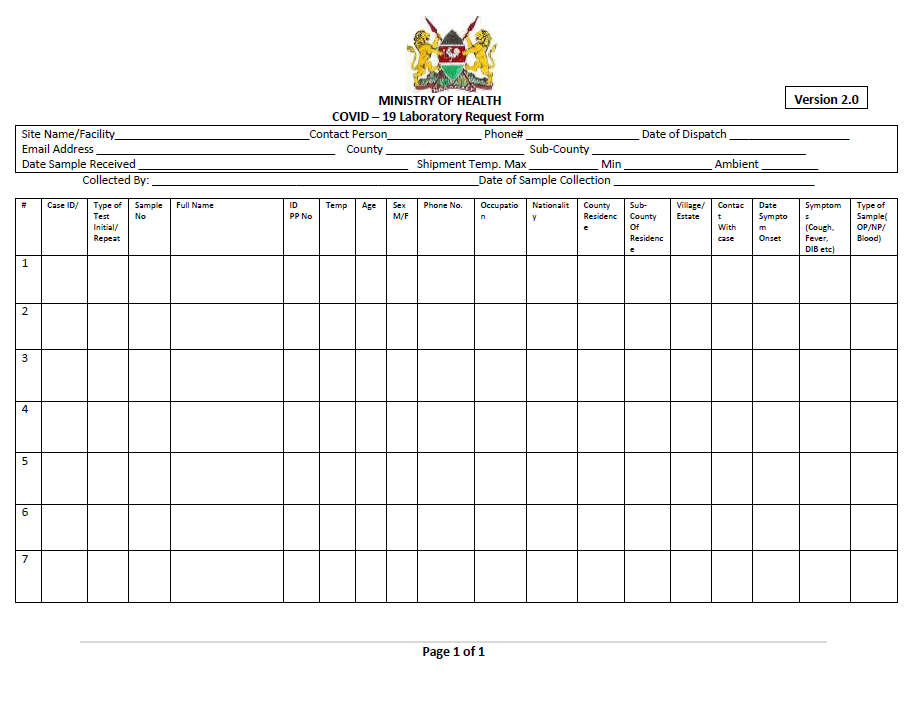


# References

References

1. Northern Corridor Transit and Transport Coordination Authority. Northern Corridor Roads Network [Available from: <http://www.ttcanc.org/maps.php> accessed December 17 2020.

2. Ritchie H, Ortiz-Ospina E, Beltekian D, et al. Coronavirus Pandemic (COVID-19) Published online at OurWorldInData.org2020 [Available from: <https://ourworldindata.org/coronavirus> accessed 18 May 2021.
